# Multiple steady states and the form of response functions to antigen in a model for the initiation of T-cell activation

**DOI:** 10.1098/rsos.170821

**Published:** 2017-11-08

**Authors:** Alan D. Rendall, Eduardo D. Sontag

**Affiliations:** 1 Institut für Mathematik, Johannes Gutenberg-Universität, Staudingerweg 9, D-55099 Mainz, Germany; 2 Department of Mathematics and the Center for Quantitative Biology, Rutgers University, Piscataway, NJ 08854, USA

**Keywords:** immunology, T cells, multistationarity

## Abstract

The aim of this paper is to study the qualitative behaviour predicted by a mathematical model for the initial stage of T-cell activation. The state variables in the model are the concentrations of phosphorylation states of the T-cell receptor (TCR) complex and the phosphatase SHP-1 in the cell. It is shown that these quantities cannot approach zero and that the model possesses more than one positive steady state for certain values of the parameters. It can also exhibit damped oscillations. It is proved that the chemical concentration which represents the degree of activation of the cell, that of the maximally phosphorylated form of the TCR complex, is, in general, a non-monotone function of the activating signal. In particular, there are cases where there is a value of the dissociation constant of the ligand from the receptor which produces a maximal activation of the T cell. This suggests that mechanisms taking place in the first few minutes after activation and included in the model studied in this paper suffice to explain the optimal dissociation time seen in experiments. In this way, the results of certain simulations in the literature have been confirmed rigorously and some important features which had not previously been seen have been discovered.

## 1. Introduction

In humans and other vertebrates, the immune system is of crucial importance for protecting an individual from dangers such as pathogens, toxins and cancer. (For background information on immunology, we refer to [1].) The central players in the immune system are the white blood cells (leucocytes) and it is important that these cells are able to distinguish between dangerous substances and host tissues. This is often referred to as the distinction between non-self and self. A failure to combat dangerous substances may lead to infectious diseases becoming life-threatening. On the other hand, if the immune system attacks host tissues, this can lead to autoimmune disease. The process of discrimination between self and non-self is complicated, involving numerous mechanisms. An important element of this process, which is investigated in the present paper, is the activity of the class of leucocytes called T cells. An individual T cell is supposed to recognize a particular substance (antigen) and take suitable action if that substance is dangerous. Recognition is based on the binding of the antigen to a molecule on the T-cell surface, the T-cell receptor (TCR). It is believed that the most important aspect of this process is the time the antigen remains bound before being released (the dissociation time), an idea which has been called the ‘lifetime dogma’ [2]. When it recognizes its antigen, the T cell changes its behaviour and is said to be activated. In this work, we study a mathematical model of the first few minutes of T-cell activation after the TCR binds to its antigen.

In [3], Altan-Bonnet and Germain introduced a model for the initial stage of T-cell activation. Simulations using this model gave results which fitted a number of experimental findings. On the other hand, it was too elaborate to be readily accessible to a mathematical analysis of its dynamics. In [4], the authors introduced a radically simplified version of the model of [3]. The new model includes the essential explanatory power of the old one while being much more transparent and tractable for analytical investigation. It also predicts features of experimental data which had not been explained previously, such as the fact that the response of a T cell can decrease as a function of the amount of antigen when the concentration of the phosphatase SHP-1 is sufficiently high. In [4], a number of interesting analytical calculations were performed, but the mathematical conclusions which can be drawn from these were not worked out in detail.

The relations between these two models will now be explained briefly. The TCR is associated with other proteins (CD3 and the *ζ*-chain), forming the TCR complex. These other proteins have cytoplasmic tails on which there are regions called immunoreceptor tyrosine-based activation motifs (ITAMs). Each ITAM contains two tyrosines on which it can be phosphorylated (i.e. phosphate groups can become bound to these tyrosines) separated by a few other amino acids. Phosphorylation of the ITAMs is a typical sign of T-cell activation. In the TCR complex, there are 10 ITAMs and thus a total of 20 phosphorylation sites of potential importance for the activation of the T cell. In a later step of the process, the protein ZAP-70 binds to the doubly phosphorylated ITAMs of the *ζ*-chains and itself becomes phosphorylated. There are two *ζ*-chains in the T-cell complex and each contains three ITAMs. Thus a total of six further phosphorylations are possible. The exact order in which all these sites are phosphorylated is not understood in detail and so this part of the system is treated in a rather schematic way in the models. In the model of [4], it is assumed that there are *N* sites which are phosphorylated sequentially, i.e. in a particular order. ZAP-70 is not included in the model. In the simulations, the choice *N*=5 is made. In the model of [3], the phosphorylation sites included are those of one *ζ*-chain and ZAP-70, leading to a total of nine. Both models include a negative feedback acting through the phosphatase SHP-1, which can dephosphorylate the sites just discussed. The importance of SHP-1 in controlling T-cell activation was pointed out in [5].

The other main difference between the models of [3,4] is the treatment of events downstream of the process of phosphorylation of the receptor complex. In [3], phosphorylation of ZAP-70 leads to a chain of events culminating in the activation by double phosphorylation of extracellular signal-regulated kinase (ERK). There is also a positive feedback loop from ERK through SHP-1 to the receptor complex. The positive feedback loop is absent from [4] and is thus seen to be unnecessary for explaining the main effects studied in [3]. In [3], it was found that the reactions linking phosphorylation of the receptor to the activation of ERK act as a switch: when the concentration of the phosphorylated receptor complex exceeds a certain threshold, ERK becomes activated. In the model of [4], this switch is incorporated in the form that when the concentration of the maximally phosphorylated form of the receptor complex exceeds a certain threshold, this is taken as the defining property of the T cell being activated.

The aim of the present paper is to obtain results about the qualitative behaviour of solutions of the model of [4] which are as general as possible. In §2, the model is defined and some of its basic properties are derived. The model describes a situation where both an agonist (the antigen which should be recognized) and an antagonist (a competing antigen) are present. Section 3 is concerned with the number of steady states and their stability. After some general results have been derived, the discussion turns to more detailed properties of the solutions in the case that the antagonist is absent and treats cases where the number *N* of phosphorylation sites included in the model is small. In particular, it is shown that when *N*=3, there are parameters for which three positive steady states exist (theorem 3.1). A numerical calculation reveals that for a specific choice of these parameters two of the steady states are stable, while the third is a saddle. For *N*≤2, there is a unique steady state and in the case *N*=1 it is proved to be globally asymptotically stable. There are parameter values for which the approach to this steady state is oscillatory.

The qualitative behaviour of the steady-state concentration of the maximally phosphorylated state, which expresses the degree of activation of the T cell, as a function of the antigen concentration and the dissociation time, is investigated in the case where only the agonist is present in §4. Let us consider the function *f*(*L*_1_,*ν*_1_), which expresses the degree of activation in terms of the parameters *L*_1_ (concentration of agonist ligand) and *ν*_1_ (reaction rate for the dissociation of the ligand from the receptor, i.e. the reciprocal of the dissociation time). It is shown that the dependence exhibits certain types of non-monotone behaviour in some cases. The results obtained include both rigorous results on general features of the function *f* (theorem 4.2) and simulations which reveal more detailed features. In particular, it is found that are values of the parameters in the model for which the function *f* has a maximum as a function of *ν*_1_ for fixed *L*_1_. In other words, there is a value of the dissociation time which is optimal for T-cell activation. Thus, the model studied here is able to reproduce this fact which has been experimentally observed [6].

The analysis of the response function is extended to cover the effects of the antagonist in §5. The last section is devoted to conclusions and an outlook.

## 2. Definition of the model

In the introduction, it was stated that a T cell recognizes an antigen. In more detail, the molecule concerned is a peptide (a small protein) which is bound to a host molecule called a major histocompatibility complex (MHC) molecule. Thus, we talk about a pMHC complex as the object to be recognized. In the model of [4], two types of pMHC complexes are considered. The first, called an agonist, represents the case where the antigen comes from a pathogen and should activate the T cell. The second, called an antagonist, represents the case of a self-antigen, which should not activate the T cell. Detection takes place through the binding of a pMHC complex to the TCR. As explained in the introduction, when this happens certain proteins associated with the TCR are phosphorylated, i.e. phosphate groups become attached to them. For simplicity, we describe this by saying that the receptor-pMHC complex is phosphorylated.

**Figure 1.**
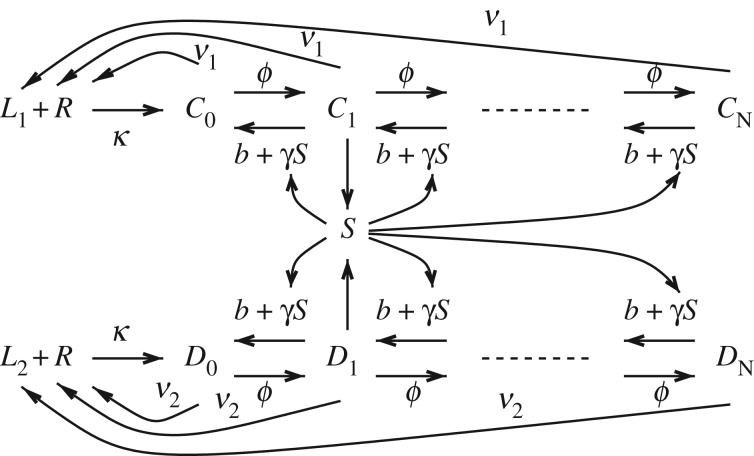
The model considered in this paper. The species *R* is the T-cell receptor (TCR), and *L*_1_ and *L*_2_ are the two ligands, i.e. the agonist and antagonist. The species *C*_0_ are unphosphorylated complexes of the TCR with the agonist, and the *C*_*j*_’s are the *j*-phosphorylated complexes. The *D*_*j*_’s are the analogous complexes for the antagonist. The phosphatase SHP-1 provides a negative feedback, and is represented by *S*. The different reactions represent receptor complex phosphorylation with rate constant *ϕ* and dephosphorylation with rate constant *b*, as well as receptor complex dephosphorylation by *S* with rate constant *γ* and dissociation rate constants *ν*_1_ and *ν*_2_. Antigens bind to *R* with rate constant *κ*, and *S* is activated by the singly phosphorylated complexes with rate constant *α* and deactivated with rate constant *β*.

The reaction network for the model of [4] is shown in figure 1. The state variables will now be listed. The concentration of unphosphorylated complexes of the TCR with the agonist is denoted by *C*_0_ and the concentration of unphosphorylated complexes of the TCR with the antagonist is denoted by *D*_0_. *C*_*j*_ and *D*_*j*_ are the corresponding quantities for the case of *j* phosphorylations, up to a maximum value *N*. The specific value of *N* has little influence in what follows. In some of our results, we choose *N* small so as to obtain the simplest possible mathematical setting. The number of phosphorylation sites relevant to the models of [3,4] have been discussed in the introduction. *R*, *L*_1_ and *L*_2_ are the total concentrations of receptors and the two ligands, i.e. the agonist and antagonist. Another important element of the system is SHP-1. This substance is a phosphatase which means that when active it can remove phosphate groups from the receptor-pMHC complex. It contributes a negative feedback loop to the system. *S* is the concentration of active SHP-1. The receptor complexes are subject to phosphorylation with rate constant *ϕ* and dephosphorylation with rate constant *b*. They are also dephosphorylated by SHP-1 with rate constant *γ* and dissociate with rate constants *ν*_1_ and *ν*_2_. Antigens bind to the receptor with rate constant *κ*. SHP-1 is activated by the singly phosphorylated complexes with rate constant *α* and deactivated with rate constant *β*. All the rate constants are assumed positive. *S*_T_ is the total concentration of SHP-1. It is assumed that all reactions exhibit mass action kinetics and this leads to the following system of equations:

2.1 $$\begin{array}{r} \dot{S}=\alpha (C_{1}+D_{1})(S_{T}-S)-\beta S, \end{array}$$

2.2 $$\begin{array}{r} {\dot{C}}_{0}=\kappa \left(L_{1}-\sum_{j=0}^{N}C_{j}\right)\left(R-\sum_{j=0}^{N}(C_{j}+D_{j})\right)+(b+\gamma S)C_{1}-(\phi +\nu_{1})C_{0}, \end{array}$$

2.3 $$\begin{array}{r} {\dot{C}}_{j}=\phi C_{j-1}+(b+\gamma S)C_{j+1}-(\phi +b+\gamma S+\nu_{1})C_{j},\;1\leq j\leq N-1, \end{array}$$

2.4 $$\begin{array}{r} {\dot{C}}_{N}=\phi C_{N-1}-(b+\gamma S+\nu_{1})C_{N}, \end{array}$$

2.5 $$\begin{array}{r} {\dot{D}}_{0}=\kappa \left(L_{2}-\sum_{j=0}^{N}D_{j}\right)\left(R-\sum_{j=0}^{N}(C_{j}+D_{j})\right)+(b+\gamma S)D_{1}-(\phi +\nu_{2})D_{0}, \end{array}$$

2.6 $$\begin{array}{r} {\dot{D}}_{j}=\phi D_{j-1}+(b+\gamma S)D_{j+1}-(\phi +b+\gamma S+\nu_{2})D_{j},\;1\leq j\leq N-1 \end{array}$$

2.7 $$\begin{array}{r} \;\text{and}\;{\dot{D}}_{N}=\phi D_{N-1}-(b+\gamma S+\nu_{2})D_{N}. \end{array}$$

In a direct formulation of the system as arising from the reaction network, it is necessary to include the concentrations of free ligands, free receptors and inactive phosphatase. This extended system has four conservation laws corresponding to the total amounts of ligands, receptors and phosphatase. The explicit form of the conserved quantities is

$$\begin{array}{rl} \sum_{j=0}^{N}C_{j}+L_{1,U} & =L_{1},\;\sum_{j=0}^{N}D_{j}+L_{2,U}=L_{2},\\ \sum_{j=0}^{N}C_{j}+\sum_{j=0}^{N}D_{j}+R_{U} & =R,\;S+S_{I}=S_{T}, \end{array}$$

where *L*_1,*U*_, *L*_2,*U*_ and *R*_*U*_ are the concentrations of unbound ligands and receptors and *S*_*I*_ is the concentration of the inactive form of SHP-1. Using these conservation laws to eliminate the additional variables leads to the system (2.1)–(2.7).

The right-hand sides of the equations are Lipschitz and so there is a unique solution corresponding to each choice of initial data. To have a biologically relevant solution, the quantities in the extended system should be non-negative. It is a well-known fact for reaction networks of this type that data for which all concentrations are positive give rise to solutions with the same property and that data for which all concentrations are non-negative give rise to non-negative solutions. In terms of (2.1)–(2.7), this implies statements about the positivity of the quantities *S*, *C*_*j*_ and *D*_*j*_ and of the differences *S*_T_−*S*, $R-\sum_{j=0}^{N}(C_{j}+D_{j})$, $L_{1}-\sum_{j=0}^{N}C_{j}$ and $L_{2}-\sum_{j=0}^{N}D_{j}$. Let us call the region where all these quantities are strictly positive the biologically feasible region. Note that owing to the conservation laws, this region is bounded. Let $\Sigma_{1}=\sum_{j=0}^{N}C_{j}$ and $\Sigma_{2}=\sum_{j=0}^{N}D_{j}$. Then it follows from (2.1) to (2.7) that

2.8 $${\dot{\Sigma }}_{1}=\kappa (L_{1}-\Sigma_{1})(R-\Sigma_{1}-\Sigma_{2})-\nu_{1}\Sigma_{1}$$

and

2.9 $${\dot{\Sigma }}_{2}=\kappa (L_{2}-\Sigma_{2})(R-\Sigma_{1}-\Sigma_{2})-\nu_{2}\Sigma_{2}.$$

**Lemma 2.1**

*Consider a solution* (*S*(*t*),*C*_0_(*t*),…,*C*_*N*_(*t*),*D*_0_(*t*),…,*D*_*N*_(*t*)) *in the closure of the biologically feasible region. Then if* $\left(S^{*},C_{0}^{*},\ldots ,C_{N}^{*},D_{0}^{*},\ldots ,D_{N}^{*}\right)$ *is an ω-limit point of this solution it is also in the biologically feasible region. In particular, any steady state is in the biologically feasible region*.

**Proof.**

If S*=*S*_T_ we can consider the solution starting at that point at some time *t*_0_. Since the *ω*-limit set of a given solution is invariant, the solution under consideration lies entirely in the *ω*-limit set of the original solution. In particular, it is contained in the closure of the biologically feasible region. The solution starting at the point with S*=*S*_T_ satisfies $\dot{S}(t_{0})<0$ because the first term on the right-hand side of (2.1) is zero for *t*=*t*_0_ and the second term negative. Hence, the solution starting at the *ω*-limit point satisfies the inequality *S*(*t*)>*S*_T_ for *t* slightly less than *t*_0_, a contradiction to the fact that the original solution was in the biologically feasible region. In a similar way, equation (2.8) implies that $\sum_{j=0}^{N}C_{j}^{*}$ cannot attain the value *L*_1_ and equation (2.9) implies that $\sum_{j=0}^{N}D_{j}^{*}$ cannot attain the value *L*_2_. Summing (2.8) and (2.9) shows that $\sum_{j=0}^{N}C_{j}^{*}+\sum_{j=0}^{N}D_{j}^{*}$ cannot attain the value *R*.

Note next that *C*_0_ cannot be zero at an *ω*-limit point. For if it is zero at such a point, we can consider the solution passing through that point at a time *t*_0_. As the inequalities already proved imply that the first term in equation (2.2) is positive for *t*=*t*_0_ that equation implies that ${\dot{C}}_{0}(t_{0})>0$ and that *C*_0_(*t*)<0 for *t* slightly less than *t*_0_, a contradiction. Once the positivity of *C*_0_ has been proved we can use equation (2.3) with *j*=1 to show that *C*_1_ cannot be zero at an *ω*-limit point. This, in turn, allows us to prove using (2.1) that *S* can never be zero at an *ω*-limit point. In a similar way, it can be concluded successively that *C*_2_,…,*C*_*N*_ and *D*_0_,…,*D*_*N*_ are positive at any *ω*-limit point of a non-negative solution. This concludes the proof of the lemma. ▪

The fact that all *ω*-limit points of solutions in the closure of the biologically feasible region are in the biologically feasible region, together with the fact that the closure of that region is compact, implies that the infimum of the distance of a given solution to the boundary in the limit t→∞ is strictly positive. When this last property holds, the system is said to be persistent [7]. Note in addition that the closure of the biologically feasible region is convex and hence homeomorphic to a closed ball in a Euclidean space. It follows from the Brouwer fixed point theorem that a steady state exists (cf. [8], ch. I, theorem 8.2). As steady states on the boundary have already been excluded, we can conclude that there is at least one steady state in the biologically feasible region for any fixed choice of parameters. That this is the case was assumed implicitly in [4].

## 3. Multiplicity of steady states

A question not addressed in [4] is whether there might exist more than one positive steady state for a fixed choice of parameters. In this section, it is shown that for some values of *N* and the reaction constants this is the case. The aim is to find any parameter values with this property while not worrying for the moment how biologically relevant this choice of parameters is. Let *f*_1_ and *f*_2_ denote the right-hand sides of equations (2.8) and (2.9). Then ∂*f*_1_/∂*Σ*_2_ and ∂*f*_2_/∂*Σ*_1_ are negative and hence the system (2.8)–(2.9) is competitive. It follows that every solution of this system converges to a steady state as t→∞ [9].

A steady state $\left(\Sigma_{1}^{*},\Sigma_{2}^{*}\right)$ of (2.8)–(2.9) satisfies the equations

3.1 $$\kappa (L_{1}-\Sigma_{1}^{*})(R-\Sigma_{1}^{*}-\Sigma_{2}^{*})-\nu_{1}\Sigma_{1}^{*}=0$$

and

3.2 $$\kappa (L_{2}-\Sigma_{2}^{*})(R-\Sigma_{1}^{*}-\Sigma_{2}^{*})-\nu_{2}\Sigma_{2}^{*}=0.$$

We can solve for $\Sigma_{1}^{*}$ and $\Sigma_{2}^{*}$ as functions of $\Sigma_{1}^{*}+\Sigma_{2}^{*}$:

3.3 $$\Sigma_{1}^{*}=\frac{\kappa L_{1}(R-\Sigma_{1}^{*}-\Sigma_{2}^{*})}{\kappa (R-\Sigma_{1}^{*}-\Sigma_{2}^{*})+\nu_{1}}$$

and

3.4 $$\Sigma_{2}^{*}=\frac{\kappa L_{2}(R-\Sigma_{1}^{*}-\Sigma_{2}^{*})}{\kappa (R-\Sigma_{1}^{*}-\Sigma_{2}^{*})+\nu_{2}}.$$

Hence

3.5 $$\kappa (L_{1}+L_{2}-\Sigma_{1}^{*}-\Sigma_{2}^{*})=\frac{\kappa L_{1}\nu_{1}}{\kappa (R-\Sigma_{1}^{*}-\Sigma_{2}^{*})+\nu_{1}}+\frac{\kappa L_{2}\nu_{2}}{\kappa (R-\Sigma_{1}^{*}-\Sigma_{2}^{*})+\nu_{2}}.$$

The function of $\Sigma_{1}^{*}+\Sigma_{2}^{*}$ on the left-hand side of this equation is decreasing on the interval [0,*L*_1_+*L*_2_]. The function on the right-hand side is increasing on the interval [0,*R*]. Their graphs can intersect in at most one point. We already know that they must intersect since a positive steady state of the full system exists. That they intersect can also be seen directly. For in all cases, the left-hand side is greater than the right-hand side for $\Sigma_{1}^{*}+\Sigma_{2}^{*}=0$ and the opposite inequality holds for $\Sigma_{1}^{*}+\Sigma_{2}^{*}=\min\{L_{1}+L_{2},R\}$. Thus, the equation has a unique solution for $\Sigma_{1}^{*}+\Sigma_{2}^{*}$ in the interval $[0,\min\{L_{1}+L_{2},R\}].$ From this, it is possible to compute values of $\Sigma_{1}^{*}$ and $\Sigma_{2}^{*}$ which solve (3.1) and (3.2) and lie in the intervals $\left[0,\min\{L_{1},R\}\right]$ and $\left[0,\min\{L_{2},R\}\right]$, respectively. The quantities $\Sigma_{1}^{*}$ and $\Sigma_{2}^{*}$ are functions of the parameters *R*, *L*_1_, *L*_2_, *κ*, *ν*_1_ and *ν*_2_.

It can be concluded that the solution passing through an *ω*-limit point of a solution of the original system satisfies a simplified system containing $\Sigma_{1}^{*}$ and $\Sigma_{2}^{*}$ as parameters. *C*_0_ and *D*_0_ can be eliminated from this system in favour of the other *C*_*j*_ and *D*_*j*_. The result is

3.6 $$\begin{array}{r} \dot{S}=\alpha (C_{1}+D_{1})(S_{T}-S)-\beta S, \end{array}$$

3.7 $$\begin{array}{r} {\dot{C}}_{1}=\phi \Sigma_{1}^{*}+(b+\gamma S-\phi )C_{2}-(2\phi +b+\gamma S+\nu_{1})C_{1}-\phi \sum_{j=3}^{N}C_{j}, \end{array}$$

3.8 $$\begin{array}{r} {\dot{C}}_{j}=\phi C_{j-1}+(b+\gamma S)C_{j+1}-(\phi +b+\gamma S+\nu_{1})C_{j},\;2\leq j\leq N-1, \end{array}$$

3.9 $$\begin{array}{r} {\dot{C}}_{N}=\phi C_{N-1}-(b+\gamma S+\nu_{1})C_{N}, \end{array}$$

3.10 $$\begin{array}{r} {\dot{D}}_{1}=\phi \Sigma_{2}^{*}+(b+\gamma S-\phi )D_{2}-(2\phi +b+\gamma S+\nu_{2})D_{1}-\phi \sum_{j=3}^{N}D_{j}, \end{array}$$

3.11 $$\begin{array}{r} {\dot{D}}_{j}=\phi D_{j-1}+(b+\gamma S)D_{j+1}-(\phi +b+\gamma S+\nu_{2})D_{j},\;2\leq j\leq N-1 \end{array}$$

3.12 $$\begin{array}{r} \;\text{and}\;{\dot{D}}_{N}=\phi D_{N-1}-(b+\gamma S+\nu_{2})D_{N}. \end{array}$$

This form of the equations is valid for *N*≥3. In the case *N*=2, it is still correct if it is taken into account that the condition 2≤*j*≤*N*−1 is never satisfied so that the equations containing that condition are absent. The sum from *j*=3 to *N* is zero in that case. The case *N*=1 is exceptional from the point of the notation.

To get more information, we restrict in the remainder of this section to, what we call, the agonist-only case. This is obtained from the system (2.1)–(2.7) by setting *L*_2_ and the *D*_*i*_ to zero. There is a corresponding limiting system, which is obtained from (3.6) to (3.12) by setting $\Sigma_{2}^{*}$ and the *D*_*i*_ to zero. In this case, we write Σ* instead of $\Sigma_{1}^{*}$ for brevity. Consider the limiting system in the agonist-only case with *N*=1. This is

3.13 $$\dot{S}=\alpha C_{1}(S_{T}-S)-\beta S$$

and

3.14 $${\dot{C}}_{1}=\phi \Sigma^{*}-(\phi +b+\gamma S+\nu_{1})C_{1}.$$

Solving the equation $\dot{S}=0$ for *C*_1_ and substituting the result into the equation ${\dot{C}}_{1}=0$ gives the quadratic equation

3.15 $$\beta \gamma S^{2}+[\beta (\phi +b+\nu_{1})+\alpha \phi \Sigma^{*}]S-\alpha \phi \Sigma^{*}S_{T}=0.$$

As the quadratic polynomial has positive leading term and is negative for *S*=0, it is clear that it has a unique positive root. It follows from (3.15) that this root is less than *S*_T_. Equation (3.14) implies that *C*_1_<Σ* at a steady state and so these quantities can be completed to a steady state of the original system by defining *C*_0_=Σ*−*C*_1_. The steady state is unique in this case.

In the case *N*=2, the equations are

3.16 $$\begin{array}{r} \dot{S}=\alpha C_{1}(S_{T}-S)-\beta S, \end{array}$$

3.17 $$\begin{array}{r} {\dot{C}}_{1}=\phi \Sigma^{*}-(2\phi +b+\gamma S+\nu_{1})C_{1}+(-\phi +b+\gamma S)C_{2} \end{array}$$

3.18 $$\begin{array}{r} \;\text{and}\;{\dot{C}}_{2}=\phi C_{1}-(b+\gamma S+\nu_{1})C_{2}. \end{array}$$

Proceeding in a manner analogous to what we did in the case *N*=1 it is possible to get a cubic equation for *S* in the case *N*=2, which we can write schematically in the form $p(S)=\sum_{k=0}^{N}a_{k}S^{k}$. We have

$$\begin{array}{rl} a_{0} & =-\alpha S_{T}(b+\nu_{1})\phi \Sigma^{*},\\ a_{1} & =\beta [b(\phi +b+\nu_{1})+\nu_{1}(2\phi +b+\nu_{1})+\phi^{2}]+\alpha (b+\nu_{1})\phi \Sigma^{*}-\alpha \gamma S_{T}\phi \Sigma^{*},\\ a_{2} & =\beta \gamma (\phi +2b+2\nu_{1})+\alpha \gamma \phi \Sigma^{*},\\ a_{3} & =\beta \gamma^{2}. \end{array}$$

The sequence of signs of the coefficients *a*_*i*_ is either (−,−,+,+) or (−,+,+,+). There is precisely one change of sign and thus by Descartes’ rule of signs the polynomial has precisely one positive root. Once a value of *S* is given, the values of *C*_1_ and *C*_2_ at the steady state can be determined successively. Following the arguments in the case *N*=1, we see that *S*<*S*_T_, *C*_1_+*C*_2_<Σ* and that the steady state is unique.

In the case *N*=3, the system is

3.19 $$\begin{array}{r} \dot{S}=\alpha C_{1}(S_{T}-S)-\beta S, \end{array}$$

3.20 $$\begin{array}{r} {\dot{C}}_{1}=\phi \Sigma^{*}-(2\phi +b+\gamma S+\nu_{1})C_{1}+(-\phi +b+\gamma S)C_{2}-\phi C_{3}, \end{array}$$

3.21 $$\begin{array}{r} {\dot{C}}_{2}=\phi C_{1}-(\phi +b+\gamma S+\nu_{1})C_{2}+(b+\gamma S)C_{3} \end{array}$$

3.22 $$\begin{array}{r} \;\text{and}\;{\dot{C}}_{3}=\phi C_{2}-(b+\gamma S+\nu_{1})C_{3}. \end{array}$$

A calculation for *N*=3 analogous to those already done gives a quartic polynomial. Its coefficients are

$$\begin{array}{rl} a_{0} & =-[{\left(b+\nu_{1}\right)}^{2}+\phi \nu_{1}]\alpha \phi \Sigma^{*}S_{T},\\ a_{1} & =\beta \gamma \{(\phi +b+\nu_{1})[(b(b+\nu_{1})+\nu_{1}(\phi +b+\nu_{1})]+\nu_{1}(\phi +b+\nu_{1})\\ & \;+\phi^{2}(b+\nu_{1})+\phi^{3}\}+[{\left(b+\nu_{1}\right)}^{2}+\nu_{1}\phi ]\alpha \phi \Sigma^{*}-2(b+\nu_{1})\alpha \gamma \phi \Sigma^{*}S_{T},\\ a_{2} & =\beta \gamma \{b(b+\nu_{1})+\nu_{1}(\phi +b+\nu_{1})+2(\phi +b+\nu_{1})(b+\nu_{1})+\phi \nu_{1}+\phi^{2}\}\\ & \;+2(b+\nu_{1})\alpha \gamma \phi \Sigma^{*}-\gamma^{2}\alpha \phi \Sigma^{*}S_{T},\\ a_{3} & =\beta \{2\gamma (b+\nu_{1})+\gamma^{2}(\phi +b+\nu_{1})\}+\gamma^{2}\alpha \phi \Sigma^{*},\\ a_{4} & =\beta \gamma^{3}. \end{array}$$

The coefficient *a*_0_ is negative, while *a*_3_ and *a*_4_ are positive. Unless *a*_1_>0 and *a*_2_<0 Descartes’ rule of signs implies that the polynomial only has one positive root. Otherwise, the rule implies that it has one or three positive roots (counting multiplicity), but does not decide between these two cases.

It will now be shown that in the case *N*=3, there are values of the coefficients for which the polynomial *p*(*S*) has three positive roots. To do this, we vary the coefficients *S*_T_ and *ν*_1_ in the system (3.19)–(3.22) and keep all others fixed. Note that these coefficients come from the parameters in the agonist-only case of (2.1)–(2.4). To obtain the desired variation of the coefficients, we fix all parameters in (2.1)–(2.4) except *S*_T_, *ν*_1_ and *κ* and vary *κ* in such a way that *ν*_1_/*κ* does not change. This ensures that Σ* does not change. In fact, we may simplify the calculations by setting *b*=0 because if three positive roots can be obtained in that case the same thing can be obtained for *b* small and positive by continuity. Suppose that *S*_T_ and *ν*_1_ depend on a parameter *ϵ* with both of them being positive for *ϵ*>0. Suppose in addition that in the limit *ϵ*→0, we have the asymptotic relations $S_{T}={\bar{S}}_{T}\epsilon^{-1}+o(\epsilon^{-1})$ and $\nu_{1}={\bar{\nu }}_{1}\epsilon^{4}+o(\epsilon^{4})$ for constants ${\bar{S}}_{T}$ and ${\bar{\nu }}_{1}$. Then we obtain asymptotic expansions *a*_4_=*A*_4_, *a*_3_=*A*_3_+*o*(1), *a*_1_=*A*_1_+*o*(1) for positive constants *A*_4_, *A*_3_ and *A*_1_, *a*_0_=*A*_0_*ϵ*^3^+*o*(*ϵ*^3^) for a constant *A*_0_<0 and *a*_2_=*A*_2_*ϵ*^−1^+*o*(*ϵ*^−1^) for a constant *A*_2_<0. Let *q*(*S*)=*ϵp*(*S*). Then *q*(1) converges to *A*_2_ for *ϵ*→0 and is thus negative for *ϵ* small enough. The same is true for *p*(1). On the other hand,

3.23 $$p(\epsilon^{2})=A_{0}\epsilon^{3}+A_{1}\epsilon^{2}+A_{2}\epsilon^{3}+A_{3}\epsilon^{6}+A_{4}\epsilon^{8}+o(\epsilon^{2})=A_{1}\epsilon^{2}+o(\epsilon^{2}).$$

Hence for *ϵ* sufficiently small *p*(*ϵ*^2^)>0. Putting these facts together shows that *p* has three positive roots when *ϵ* is small. For each of these roots, the values of *C*_1_, *C*_2_ and *C*_3_ at the steady state can be determined successively. *S*<*S*_T_, *C*_1_+*C*_2_+*C*_3_<Σ* and defining *C*_0_=Σ*−(*C*_1_+*C*_2_+*C*_3_) gives a steady state of the original system.

It has already been noted that *p* cannot have more than three positive roots. There are parameter values for which the positive steady state is unique. To see this, it is enough to assume that *S*_T_ is small while keeping the other parameters fixed. Then *a*_*i*_>0 for all *i*>0 and the polynomial can have no more that one positive root because its derivative has no positive root. These results can be summed up as follows:

**Theorem 3.1**

*The agonist-only case of the system* (*2.1*)–(*2.7*) *has exactly one positive steady state for N*=1 *and N*=2. *In the case N*=3, *there are parameters for which it has three positive steady states and it can never have more than three*.

A concrete example of parameters for which there are three positive steady states is obtained by setting *α*, *β*, *γ*, *ϕ*, *L*_1_ and *R* equal to one and choosing *S*_T_=10, *κ*=2×10^−4^, *ν*_1_=10^−4^. A computer calculation shows that the coordinates $\left(S^{*},C_{0}^{*},C_{1}^{*},C_{2}^{*},C_{3}^{*}\right)$ of the steady states are approximately

3.24 $$\begin{array}{r} (1.1769,0.1570,0.1334,0.1133,0.0963), \end{array}$$

3.25 $$\begin{array}{r} \left(0.0005,0.0001,0.0001,0.0003,0.4996\right) \end{array}$$

3.26 $$\begin{array}{r} \;\text{and}\;(0.2860,0.0085,0.0294,0.1028,0.3593). \end{array}$$

**Figure 2.**
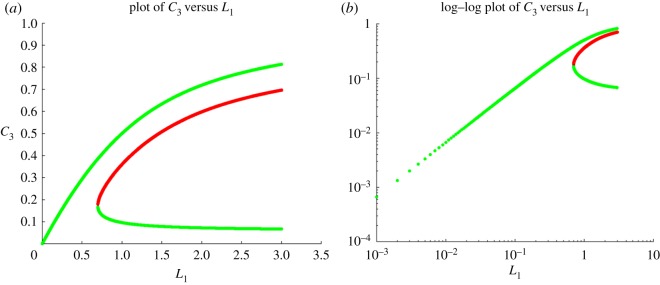
Multistability of steady states as a function of *L*_1_. Shown is the coordinate *C*_3_, but other coordinates behave similarly. Stable branches are shown in green and unstable in red. (*a*) Linear scale, (*b*) log–log scale. Parameters are *α*=1, *S*_T_=10, *β*=1, *κ*=2×10^−4^, *R*=1, *b*=0, *γ*=1, *ϕ*=1, *ν*_1_=1.

It shows in addition that while the first and second of these steady states are asymptotically stable the third is a saddle with a one-dimensional unstable manifold. A plot of the steady states as a function of the parameter *L*_1_ (figure 2), suggests that there is a fold bifurcation.

For higher values of *N* it is possible to derive a polynomial equation of degree *N*+1 for *S*. There is no obvious reason why this polynomial should not have an arbitrarily large number of positive roots for *N* arbitrarily large. A simple upper bound is that the polynomial can have no more than *N* positive roots for *N* odd and no more than *N*+1 for *N* even. Simulations indicate that in the case *N*=5 there are parameters for which three steady states exist but no parameters were found for which there are more than three for any value of *N*.

In general, it is difficult to obtain information about the stability of the steady states by analytic methods. In the case *N*=1, the vector field defining the dynamical system has negative divergence and so by Dulac’s criterion und Poincaré–Bendixson theory, all solutions converge to the steady state as t→∞. The system can exhibit damped oscillations as will now be shown. To do this, we choose parameters so that

3.27 $$\alpha C_{1}+\beta =\phi +b+\gamma S+\nu_{1}.$$

For fixed values of the quantities *R* and *S*_T_, the quantities *C*_1_ and *S* are bounded uniformly in the quantities appearing in (3.27). Thus, if we make *α* and *β* small while fixing the other parameters, we can arrange that the left-hand side is smaller than the right-hand side. If starting from there, we make *β* large while fixing the other parameters we can arrange that the left-hand side of (3.27) is greater than that of the right-hand side. It follows that parameter values exist for which (3.27) holds. Why this is interesting is that the discriminant of the characteristic equation of the linearization is the sum of a term which vanishes when (3.27) holds and the expression −4*αγ*(*S*_T_−*S*)*C*_1_. Thus when (3.27) holds, the linearization has eigenvalues with negative real part and non-zero imaginary part and there are damped oscillations.

An interesting limiting case of the agonist-only system is obtained by assuming that *α*=0 and *S*=0. We refer to this as the kinetic proofreading system because it is closely related to McKeithan’s kinetic proofreading model [10]. In fact, McKeithan only considered the case *b*=0, but this makes no essential difference for the analysis which follows. It was observed by Sontag [11] that the deficiency zero theorem of chemical reaction network theory can be applied to McKeithan’s system to conclude that there is a unique steady state in each stoichiometric compatibility class and that this solution is asymptotically stable in its class. Strictly speaking, chemical reaction network theory is applied to the extended system which includes free receptors and free ligand as variables. To show that the steady state is globally asymptotically stable, it suffices to show that there are no *ω*-limit points on the boundary. That this is the case can be proved just as we did for the full system above. The steady state is hyperbolic as follows from appendix C of [12].

Consider now the full agonist-only system. Setting *α*=0 gives a system where the kinetic proofreading system is coupled to a system describing the decay of *S*. The steady state of the kinetic proofreading system gives rise to a steady state of the agonist-only system with *α*=0 which is on the boundary of the biologically feasible region and is a hyperbolic sink. Denote its coordinates by $\left(0,C_{j}^{*}\right)$. For *α* small and positive, there exists a hyperbolic sink which is a small perturbation of that for *α*=0. It must be in the biologically feasible region because *C*_1_>0 there and equation (2.1) would imply that $\dot{S}>0$ there if *S* were negative. Thus for sufficiently small values of *α*, there exists a positive steady state which is a hyperbolic sink $\left(S^{*}(\alpha ),C_{j}^{*}(\alpha )\right)$ close to $\left(0,C_{j}^{*}\right)$. There exists a positive number *r* such that for *α* sufficiently small, say *α*≤*α*_0_, $\left(S^{*}(\alpha ),C_{j}^{*}(\alpha )\right)$ is the only *ω*-limit point of any solution in the open ball of radius *r* about that steady state.

Let *h*(*C*_*j*_) be the Lyapunov function in the proof of the deficiency zero theorem. It is known from general arguments that $\dot{h}\leq 0$ with equality only for $C_{j}=C_{j}^{*}$. It follows that on the complement of the ball of radius *r* about the steady state the function $\dot{h}$ has a strictly negative maximum. We can consider the behaviour of the function $\dot{h}$ for solutions of the system for positive *α*. For small *α*, it is still a Lyapunov function on the complement of a small ball about the steady state, while there are no *ω*-limit points except the steady state itself within that ball. Hence for *α*, sufficiently small a solution can have no *ω*-limit points other than the steady state. It follows that for *α* small the steady state is globally asymptotically stable. Of course, this means that the limiting system obtained from the agonist-only system by passing to a solution through an *ω*-limit point also has a unique steady state which is globally asymptotically stable for *α* sufficiently small. A similar argument applies in the case of the full system (2.1)–(2.7) because in that case the system obtained by setting *α* and *S* to zero is just the product of two copies of the corresponding system in the agonist-only case.

## 4. The response function

This section is concerned with the agonist-only system. From a biological point of view, the essential input parameters to the system are the ligand concentration *L*_1_ and the binding time of the ligand to the receptor, which in the model corresponds to $\nu_{1}^{-1}$. The latter is a measure of the signal strength. The essential output is the value of *C*_*N*_ which is a measure of the activation of the T cell. Given values of *L*_1_, *ν*_1_ and the other parameters, we can consider the value of *C*_*N*_ in a steady state. In fact, it is more convenient to use the quantities $\log C_{N}$ and $\log L_{1}$. This leads to a response function $\log C_{N}^{*}=F(\log L_{1},\nu_{1})$. If there is more than one steady state for a given choice of the parameters, this has to be thought of as a multi-valued function. It might naively be thought that *F* should be an increasing function of *L*_1_ and a decreasing function of *ν*_1_: more antigen leads to more activation of the T cell and a longer binding time leads to more activation. This turns out not to be the case and the function *F* is not a monotone function of its arguments. This was observed in the case of the dependence on *L*_1_ in the simulations of [4]. It is possible to understand intuitively how this situation can arise. An increase in the stimulation of the T cell leads to activation of SHP-1 and that in turn has a negative effect on the activation of the T cell. Many of the calculations in this section are guided by those in [4].

The behaviour of the response function will be estimated in various parameter ranges. To do this, it is useful to parametrize the solutions in a certain manner which will now be described. In the case of a steady state, the equation (2.3) is a linear difference equation for the *C*_*j*_ with constant coefficients. This suggests looking for power-law solutions, an idea which motivates the following result.

**Lemma 4.1**

*Steady-state solutions of equations* (*2.2*)–(*2.4*) *in the agonist-only case can be parametrized in the form*

4.1 $$C_{j}=a_{+}r_{+}^{j}+a_{-}r_{-}^{j},$$

*where the coefficients r*_±_ *and a*_±_ *are positive and depend on S. The quantities r*_+_ *and r*_−_ *are given by*

4.2 $$r_{\pm }=\frac{\phi +b+\gamma S+\nu_{1}\pm \sqrt{{\left(\phi +b+\gamma S+\nu_{1}\right)}^{2}-4\phi (b+\gamma S)}}{2(b+\gamma S)}$$

*and satisfy r*_−_<1<*r*_+_.

**Proof.**

Note first that the quantities *r*_±_ in (4.2) are the roots of the characteristic equation

4.3 $$\phi +(b+\gamma S)r^{2}-(\phi +b+\gamma S+\nu_{1})r=0,$$

associated to the difference equation already mentioned and it is obvious that they are positive. The fact that they satisfy the characteristic equation is equivalent to the condition that the *C*_*j*_ defined by (4.1) satisfy the steady state form of equation (2.3). That *r*_−_<1<*r*_+_ can be seen by noting that the function on the left-hand side of (4.3) is negative at *r*=1. The condition that the quantities *C*_*j*_ satisfy the equations (2.2)–(2.4) with ${\dot{C}}_{j}=0$ is equivalent to the conditions that they satisfy (4.1) with *r*_±_ as in (4.2) and certain coefficients *a*_−_ and *a*_+_ together with the equations obtained by substituting (4.1) into the equations ${\dot{C}}_{0}=0$ and ${\dot{C}}_{N}=0$. The explicit form of these last equations is

4.4 $$[(b+\gamma S)r_{-}-(\phi +\nu_{1})]a_{-}+[(b+\gamma S)r_{+}-(\phi +\nu_{1})]a_{+}=-\nu_{1}\sum_{j=0}^{N}C_{j}$$

and

4.5 $$r_{-}^{N-1}[\phi -(b+\gamma S+\nu_{1})r_{-}]a_{-}+r_{+}^{N-1}[\phi -(b+\gamma S+\nu_{1})r_{+}]a_{+}=0.$$

It follows from the discussion in §3 that $\sum_{j=0}^{N}C_{j}$, which was denoted there by $\Sigma_{1}^{*}$, is uniquely determined for fixed values of the parameters in (2.2)–(2.4) and fixed *S*. Thus for fixed values of these parameters and *S*, all quantities in (4.4) and (4.5) except *a*_−_ and *a*_+_ are known. It will now be shown that these equations have a unique solution for *a*_−_ and *a*_+_. Note that

4.6 $$[\phi -(b+\gamma S+\nu_{1})r_{-}][\phi -(b+\gamma S+\nu_{1})r_{+}]=-\frac{\phi^{2}\nu_{1}}{b+\gamma S},$$

as can most easily be seen by multiplying out the left-hand side of this equation and substituting for *r*_+_*r*_−_ and *r*_+_+*r*_−_, which are the sum and product of the roots of the characteristic equation (4.3). Thus equation (4.5) gives a positive expression for *a*_+_/*a*_−_. Note also that (4.6) implies that the factors in the product on the left-hand side of that equation have opposite signs. As *r*_−_<*r*_+_, the first factor is positive and the second negative. Substituting the expression for *a*_+_/*a*_−_ into (4.4) gives an equation of the form

4.7 $$a_{-}\left[A-B{\left(\frac{r_{-}}{r_{+}}\right)}^{N-1}\right]=-\nu_{1}\Sigma_{1}^{*}[\phi -(b+\gamma S+\nu_{1})r_{+}],$$

whose right-hand side is positive. Here

4.8 $$A=[(b+\gamma S)r_{-}-(\phi +\nu_{1})][\phi -(b+\gamma S+\nu_{1})r_{+}]$$

and

4.9 $$B=[(b+\gamma S)r_{+}-(\phi +\nu_{1})][\phi -(b+\gamma S+\nu_{1})r_{-}].$$

It follows from the fact that the first factor on the left-hand side of (4.6) is positive that the first factor in the expression for *A* is negative and hence that *A* itself is positive. In addition, a straightforward computation shows that *A*>*B*. If *B* were not positive, then the quantity in square brackets on the left-hand side of (4.7) would be positive. If *B* is positive, then the fact that *r*_−_<*r*_+_ implies that the quantity in square brackets is again positive. Hence in any case, (4.7) can be solved to give a unique positive value of *a*_−_. Then *a*_+_ can be determined in such a way that (4.4) and (4.5) hold. This completes the proof of lemma 4.1. ▪

Lemma 4.1 shows that for fixed parameters in (2.2)–(2.4) and a fixed value of *S* the steady-state values of all the *C*_*j*_ are determined, but this does not yet give expressions for the *C*_*j*_ which can be directly applied to study the properties of the response function. For the purposes of what follows, it is convenient to rewrite (2.8) in the form

4.10 $$\kappa \left(L_{1}-\sum_{j=0}^{N}C_{j}\right)\left(R-\sum_{j=0}^{N}C_{j}\right)-\nu_{1}\sum_{j=0}^{N}C_{j}=0.$$

The equation for *S* can be solved to give the relation *S*=*S*_T_(*C*_1_/(*C*_1_+C_*_) with C_*_=*β*/*α*. Summing the expression for *C*_*j*_ given in Lemma 2 over *j* gives

4.11 $$\sum_{j=0}^{N}C_{j}=a_{+}\frac{r_{+}^{N+1}-1}{r_{+}-1}+a_{-}\frac{r_{-}^{N+1}-1}{r_{-}-1}.$$

The following equation relating *a*_−_ and *a*_+_ is equation (21) of [4]:

4.12 $$a_{+}=-a_{-}{\left(\frac{r_{-}}{r_{+}}\right)}^{N+1}\frac{r_{+}-1}{r_{-}-1}.$$

Combining the last two equations gives

4.13 $$\sum_{j=0}^{N}C_{j}=\frac{a_{-}}{1-r_{-}}\left[1-{\left(\frac{r_{-}}{r_{+}}\right)}^{N+1}\right].$$

Having completed the necessary preliminaries, we now proceed to study the qualitative behaviour of the response function in different regimes. When *L*_1_ is small, it is to be expected that the concentration of the phosphatase is small and that the response function resembles that of the kinetic proofreading model. It will now be shown that when *L*_1_ is small, the leading term in the function *F* depends linearly on $\log L_{1}$ with slope one and the additive constant in this linear function will be determined. The equation (4.10) can be written in the form

4.14 $$\sum_{j=0}^{N}C_{j}=\frac{\kappa RL_{1}}{\kappa R+\nu_{1}}\left[1+\frac{L_{1}}{R}\left({\left(\frac{\sum_{j=0}^{N}C_{j}}{L_{1}}\right)}^{2}-\left(\frac{\sum_{j=0}^{N}C_{j}}{L_{1}}\right)\right)\right].$$

Note that $\sum_{j=0}^{N}C_{j}\leq L_{1}$ so that this equation implies that

4.15 $$\sum_{j=0}^{N}C_{j}=\frac{\kappa RL_{1}}{\kappa R+\nu_{1}}\left(1+\frac{qL_{1}}{R}\right),$$

where $-\frac{1}{4}<q<0$. Using (4.12), it is possible to write down an explicit expression for *C*_*N*_, namely

4.16 $$C_{N}=\frac{a_{-}r_{-}^{N}(r_{+}-r_{-})}{r_{+}(1-r_{-})}.$$

It follows from (4.13) that

4.17 $$C_{N}=r_{-}^{N}\frac{1-r_{-}/r_{+}}{1-{\left(r_{-}/r_{+}\right)}^{N+1}}\sum_{j=0}^{N}C_{j}.$$

Combining these equations gives

4.18 $$C_{N}=\left\{r_{-}^{N}\frac{1-r_{-}/r_{+}}{1-{\left(r_{-}/r_{+}\right)}^{N+1}}\frac{\kappa R}{\kappa R+\nu_{1}}\right\}L_{1}\left(1+\frac{qL_{1}}{R}\right).$$

The function of *r*_−_ and *r*_+_ in this equation defines a function of *S*. This function of *S* tends to a positive limiting value as *S*→0. Now $C_{1}\leq \sum_{j=0}^{N}C_{i}=O(L_{1})$ and *S*=*O*(*C*_1_). Hence for *R* fixed, we can replace the function of *r*_+_ and *r*_−_ in the above expression by its limiting value for *S*→0. If the resulting relation is plotted logarithmically, it gives a straight line of slope one as the leading order approximation in the limit $\log L_{1}\to -\infty$.

Next we look at an intermediate regime where the amount of activated SHP-1 is well away from both zero and *S*_T_. As a first step, we obtain an estimate for *r*_−_ which is sharper than that in lemma 4.1. To do this, we compute the left-hand side of the characteristic equation (4.3) for *r*=*ϕ*/(*ϕ*+*ν*_1_). The result is −*ϕν*_1_(*b*+*γS*)/(*ϕ*+*ν*_1_)^2^<0. It follows that *r*_−_<*ϕ*/(*ϕ*+*ν*_1_). Hence 1−*r*_−_>*ν*_1_/(*ϕ*+*ν*_1_). Substituting this into (4.13) gives $a_{-}>(\nu_{1}/(\phi +\nu_{1}))(\sum_{j=0}^{N}C_{j})$. Note that $S/S_{T}\geq \min\{C_{1}/2C_{*},\frac{1}{2}\}$. Hence a positive lower bound for *C*_1_ implies a positive lower bound for *S*/*S*_T_.

Next, we will derive a lower bound for *γS* in the case that *S*_T_ is large. This will be proved by contradiction. Suppose that *γS*≤*ρ* for some *ρ*>0. Then it follows from the characteristic equation that *r*_−_≥*ϕ*/(*ϕ*+*ρ*+*ν*_1_). Using this in the equation for *C*_1_ gives $C_{1}\geq (\phi \nu_{1}/\left(\phi +\nu_{1})(\phi +\rho +\nu_{1}\right))(\sum_{j=0}^{N}C_{j})$. It follows that

4.19 $$S\geq S_{T}\min\left\{\frac{\phi \nu_{1}}{2C_{*}(\phi +\nu_{1})(\phi +\rho +\nu_{1})}\left(\sum_{j=0}^{N}C_{j}\right),\frac{1}{2}\right\}.$$

It is then clear that for a given value of *ρ* and fixed values of the parameters other than *S*_T_, this leads to a contradiction if *S*_T_ is sufficiently large. In other words, given any *ρ*>0 there is a lower bound for *S*_T_ which implies that *γS*≥*ρ*. It is convenient to make the restrictions that *κR*≥1 and *L*_1_/*R*≤1 since then it is possible to replace $\sum_{j=0}^{N}C_{j}$ in (4.19) by 3*L*_1_/4(1+*ν*_1_) using (4.15).

From (4.2), it can be concluded that

4.20 $$r_{-}=\frac{\phi }{b+\gamma S}(1+O(\eta ))$$

and

4.21 $$r_{+}=1+O(\eta ).$$

where *η*=(*ϕ*+*ν*_1_)/(*b*+*γS*). This gives approximate expressions for the roots of the characteristic equation if (*ϕ*+*ν*_1_)/(*b*+*γS*) is small. As a consequence of these equations

4.22 $$\frac{r_{-}}{r_{+}}=\frac{\phi }{b+\gamma S}(1+O(\eta )).$$

Taking the expression for *C*_1_ supplied by Lemma 2 and using (4.12), (4.13) and (4.15) gives

4.23 $$C_{1}=r_{-}\frac{\kappa RL_{1}}{\kappa R+\nu_{1}}(1+O(\eta )).$$

This implies that *C*_1_=*O*(*η*) and the expression relating *S* and *C*_1_ then shows that $\frac{S}{S_{T}}=O(\eta )$. In fact,

4.24 $$C_{1}=\frac{C_{*}S}{S_{T}}(1+O(\eta )).$$

These relations indicate that in leading order *r*_−_ is proportional to *S*. However, it is also the case that

4.25 $$r_{-}=\frac{1}{S}\frac{\phi }{\gamma }\frac{1}{1+b/(\gamma S)}(1+O(\eta )),$$

which indicates that in leading order *r*_−_ is proportional to *S*^−1^. Hence

4.26 $$r_{-}=\frac{C_{*}(\kappa R+\nu_{1})}{\kappa RL_{1}S_{T}}S(1+O(\eta ))$$

and

4.27 $$r_{-}=\frac{1}{S}\frac{\phi }{\gamma }(1+O(\eta^{\prime })),$$

where $\eta^{\prime }=\max\{\eta ,b/(\gamma S)\}$. Combining these two relations gives

4.28 $$S=\sqrt{\frac{\phi \kappa RS_{T}L_{1}}{C_{*}\gamma (\kappa R+\nu_{1})}}(1+O(\eta^{\prime })).$$

Substituting this back into the equation for *r*_−_ gives

4.29 $$r_{-}=\sqrt{\frac{\phi C_{*}(\kappa R+\nu_{1})}{\gamma S_{T}L_{1}\kappa R}}(1+O(\eta^{\prime })).$$

This means that

4.30 $$\begin{array}{rl} C_{N} & =\left(\sum_{j=0}^{N}C_{j}\right)r_{-}^{N}(1+O(\eta^{\prime \prime }))\\ & ={\left(\frac{\kappa R+\nu_{1}}{\kappa RL_{1}}\right)}^{N/2-1}{\left(\frac{\phi C_{*}}{\gamma S_{T}}\right)}^{N/2}(1+O(\eta^{\prime \prime }))\\ & ={\left(\frac{\phi \beta }{\alpha \gamma S_{T}}\right)}^{N/2}{\left(\frac{\kappa R+\nu_{1}}{\kappa R}\right)}^{N/2-1}{\left(L_{1}\right)}^{1-N/2}(1+O(\eta^{\prime \prime })), \end{array}$$

where $\eta^{\prime \prime }=\max\{\eta^{\prime },L_{1}/R\}$. Choosing *L*_1_ small enough makes *L*_1_/*R* small. With *L*_1_ fixed, making *S*_T_ large enough makes *η* small. Thus, *η*′′ can be made as small as desired by choosing *L*_1_ sufficiently small and *S*_T_ sufficiently large.

**Theorem 4.2**

*Consider the response function* $\log C_{N}=F(\log L_{1},\nu_{1})$ *for the steady states of the system* (*2.1*)–(*2.4*) *with L*_2_=0 *and D*_*j*_=0. *Choose fixed values for all parameters in the system except L*_1_ *and S*_T_. *Suppose that κR*≥1. *Let ϵ*>0. *Then for any sufficiently small constant δ*>0, *the following holds. If* 0<*L*_0_<*δ, there exists μ*>0 *such that if S*_T_≥*μ the inequality*

4.31 $$\left|{\left(\frac{\phi \beta }{\alpha \gamma S_{T}}\right)}^{-N/2}{\left(\frac{\kappa R+\nu_{1}}{\kappa R}\right)}^{1-N/2}{\left(L_{1}\right)}^{N/2-1}F(\log L_{1},\nu_{1})-1\right|<\epsilon ,$$

*holds on the interval* $\left[\log L_{0},\log\delta \right]$.

**Proof.**

To obtain the conclusion of the theorem, it suffices to show that under the given assumptions *η*′′ can be made as small as desired. That this is possible follows from the above discussion. ▪

Note that this theorem implies, in particular, that for *N*>2 and suitable values of *L*_1_ and *S*_T_ there exists a range of *L*_1_ in which the response function is decreasing. The theorem also implies that in this regime, the response function can be an increasing function of *ν*_1_. This effect was not captured by the calculations of [4] because there *ν*_1_/*κR* was assumed to be so small as to be negligible.

Finally, we examine the regime where *L*_1_/*R* is small, but the phosphatase is close to being completely activated. This means that *S*/*S*_T_ is close to one. This holds provided *C*_1_ is sufficiently large compared to C_*_. It remains to check that such a regime actually occurs for some values of the parameters. It is possible to make $\sum_{j=0}^{N}C_{j}$ large while keeping *L*_1_/*R* constant. This can be done by making *R* large. This makes *a*_−_ large without making *r*_−_ small. Hence it makes *C*_1_ large and hence *S* close to *S*_T_. In this regime, the function of *r*_+_ and *r*_−_ occurring in the expression for *C*_*N*_ can be replaced by its limit for *S*→*S*_T_ and we again get a region where the slope of the graph of $\log C_{N}$ as a function $\log L_{1}$ is one but the line has been shifted compared to that obtained for *L*_1_/*R* small.

**Figure 3.**
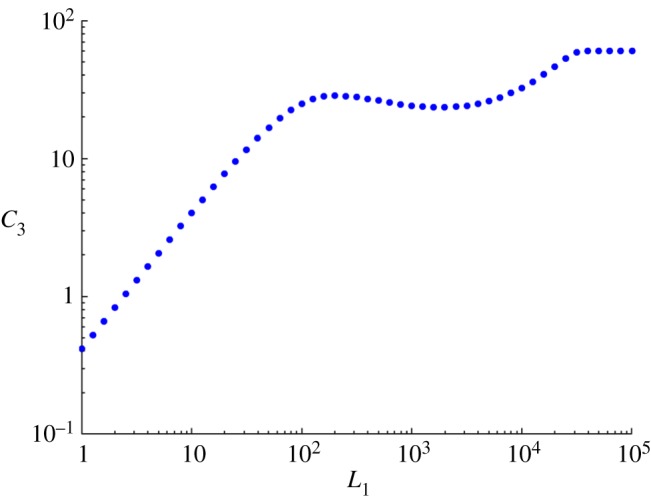
Log–log plot showing linearity of $\log C_{3}$ as a function of $\log L_{1}$ for small *L*_1_, followed by decreasing, increasing and saturation regimes. Parameters are *α*=1, *S*_T_=6×10^5^, *β*=5×10^2^, *κ*=10^−4^, *R*=3×10^4^, *b*=4×10^−2^, *γ*=1.2×10^−6^, *ϕ*=9×10^−2^, *ν*_1_=10^−2^.

In [4], these types of behaviour were exhibited numerically in the case *N*=5 with biologically reasonable choices of the parameters. We found that changing these parameters a little allows similar observations to be made in the case *N*=3. In the plot shown in figure 3, the three regimes can be seen together with a fourth regime where *L*_1_/*R* is no longer small. It is clear that a regime of this type must exist because the response function is globally bounded.

**Figure 4.**
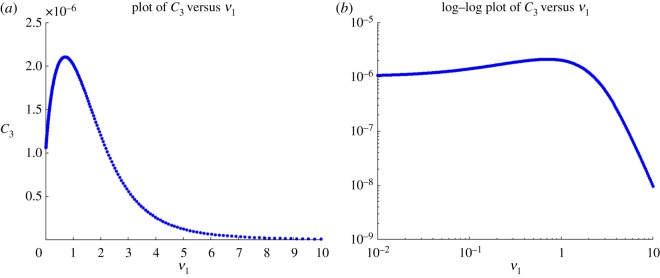
*C*_3_ as a function of *ν*_1_ in model with *N*=3, showing non-monotonic behaviour for some values of parameters. (*a*) Linear scale, (*b*) log–log scale. Parameters are *α*=10^−1^, *S*_T_=10^7^, *β*=10, *κ*=10^−6^, *R*=10^5^, *b*=10^−2^, *γ*=10^−4^, *ϕ*= 10^−2^, *L*_1_=10^3^.

We now turn to the dependence of the response function on *ν*_1_. It has been suggested in [13] that the kinetic proofreading model with negative feedback as studied here is not able to explain the presence of an optimal dissociation time, a biological effect confirmed by the experimental work of [6]. The plots of the response as a function of the dissociation time in that type of model in [13] show that it is increasing. Having an optimal dissociation time would require that there be a region where this function is decreasing. The response function being increasing as a function of the dissociation time corresponds to its being decreasing a function of *ν*_1_. Here, we have given an analytical proof in theorem 4.2 that there exist parameters for which the response is an increasing function of *ν*_1_, in contrast with the plots in [13]. As the theorem is of limited help in finding explicit parameters for which this happens, we also did a numerical search and identified parameters of this type. The results are displayed in figure 4, where it is seen that *F* has a maximum as a function of *ν*_1_ for fixed *L*_1_, which corresponds to an optimal dissociation time. The conclusion of both the analytical and the numerical work is as follows. The claim that the kinetic proofreading model with feedback can only produce a response which is a decreasing function of the parameter *ν*_1_ is dependent on the parameter values chosen to do the simulations and not a general property of the model. This means that the model of [4] can reproduce the observation of an optimal dissociation time and that as a consequence that phenomenon could arise by the mechanisms taking place in the first few minutes of activation which are included in the model of [4].

## 5. Including the antagonist

When the antagonist is included, the output variable expressing the degree of activation of the T cell is *C*_*N*_+*D*_*N*_. Now asymptotic expressions for this quantity will be derived. It has already been shown that for a steady state of the system (2.1)–(2.7), the quantities $\sum_{j=0}^{N}C_{j}$ and $\sum_{j=0}^{N}D_{j}$ can be expressed in terms of the parameters. The equation for *S* can be solved to give the relation *S*=*S*_T_((*C*_1_+*D*_1_)/(*C*_1_+*D*_1_+C_*_)). *C*_*j*_ solves the same difference equation as in the agonist-only case and *D*_*j*_ solves the difference equation obtained from that one by replacing *ν*_1_ by *ν*_2_. The quantities *r*_−_, *r*_+_, *a*_−_ and *a*_+_ differ in the two cases. We can, nevertheless, proceed as in the former case to see that the solutions for *C*_*j*_ and *D*_*j*_ allow parametrizations in terms of these quantities as before. Note that using the equations (2.8) and (2.9), it is possible to eliminate the *D*_*j*_ from the equation for *C*_0_ and the *C*_*j*_ from the equation for *D*_0_. Thus, we have coupled equations for the *C*_*j*_ and *D*_*j*_ which can be analysed just as in the agonist-only case to express *C*_1_ and *D*_1_ as functions of *S* and the parameters. We can also write *C*_*N*_ and *D*_*N*_ as functions of *Σ*_1_ and *Σ*_2_, respectively. Proceeding as in the agonist-only case, we get an expression for *C*_*N*_+*D*_*N*_ in the kinetic proofreading regime. The multiple of *L*_1_ obtained there as leading term is replaced by a linear combination of *L*_1_ and *L*_2_.

Next the intermediate regime will be considered. For this, it is necessary to define a new parameter $\eta =\max\{(\phi +\nu_{1})/(b+\gamma S),(\phi +\nu_{1})/(b+\gamma S)\}$. There are asymptotic expressions for *r*_−_ and *r*_+_ where the leading terms are just as in the agonist-only case. In particular, they are the same for *C*_*j*_ and *D*_*j*_. Two asymptotic expressions for the quantity *C*_1_+*D*_1_ can be obtained:

5.1 $$\begin{array}{r} C_{1}+D_{1}=\frac{C_{*}S}{S_{T}}(1+O(\eta )), \end{array}$$

5.2 $$\begin{array}{r} =r_{-}\left(\frac{\kappa RL_{1}}{\kappa R+\nu_{1}}+\frac{\kappa RL_{2}}{\kappa R+\nu_{2}}\right)(1+O(\eta )). \end{array}$$

This gives an expression for *r*_−_ in terms of *S*. As in the agonist-only case, this gives an expression for *r*_−_ where the dependence on *S* has been eliminated in leading order:

5.3 $$r_{-}=\sqrt{\frac{\phi C_{*}}{\gamma S_{T}}{\left(\frac{\kappa RL_{1}}{\kappa R+\nu_{1}}+\frac{\kappa RL_{2}}{\kappa R+\nu_{2}}\right)}^{-1}}(1+O(\eta )),$$

where *η*′ is defined in terms of *η* as in the agonist-only case. Following the steps used in the agonist-only case leads to an expression for *C*_*N*_+*D*_*N*_ which is the same as that previously obtained for *C*_*N*_ except that the expression *κRL*_1_/(*κR*+*ν*_1_) is replaced by *κRL*_1_/(*κR*+*ν*_1_)+*κRL*_2_/(*κR*+*ν*_2_). This leads in the end to an asymptotic expression for *C*_*N*_+*D*_*N*_ under a suitable assumption on *L*_1_ and *L*_2_. The assumption made in the agonist-only case can naturally be written as an assumption on *κRL*_1_/(*κR*+*ν*_1_) and in the present case it is replaced by an assumption on *κRL*_1_/(*κR*+*ν*_1_)+*κRL*_2_/(*κR*+*ν*_2_). This implies that under certain circumstances, *C*_*N*_+*D*_*N*_ increases when *L*_2_ increases and *L*_1_ is held fixed. An increase in the amount of self-antigen can lead to a decrease in the response to a foreign antigen. Note that the restriction needed to make this result hold is that first *L*_1_ and *L*_2_ are sufficiently small and then, with upper limits for these quantities having been fixed, that second *S*_T_ is sufficiently large. It follows that these conditions can be achieved in situations where *L*_1_/*R* and *L*_2_/*R* are as small as desired and hence the competition of the antagonist with the agonist for occupancy of the receptor is negligible. Hence the effect by which more antagonist leads to a decrease in the response to an agonist is, in general, owing to the influence of SHP-1. This gives a rigorous confirmation of a fact already observed in [4].

## 6. Conclusion and outlook

In this paper, some properties of the solutions of the model of [4] for T-cell activation were proved. A new discovery was that already in the case of three phosphorylation sites (*N*=3), there can exist more than one positive steady state for given values of the parameters. Another new observation is that damped oscillations can occur. It was also proved that, as suggested by the calculations in [4], the output variable *C*_*N*_ (concentration of the maximally phosphorylated receptor) is a decreasing function of the concentration *L*_1_ of antigen in some parts of parameter space. In an analogous way, it was proved that under some circumstances the activation in response to an agonist can be decreased by increasing the concentration of the antagonist. It was proved that it can also happen that *C*_*N*_ is an increasing function of the dissociation constant *ν*_1_. This abstract result was given a concrete illustration by a plot showing that *C*_*N*_ can have a local maximum as a function of *ν*_1_.

The stability of the steady states was only determined analytically in the very special cases *N*=1 and *α* close to zero. For *N*=3, numerical calculations showed the occurrence of two stable steady states for certain values of the parameters. It was proved that damped oscillations occur, but can there also be sustained oscillations (periodic solutions)? It is, thus, clear that there remain several aspects of the dynamics of this system which would profit from further investigations, analytical and numerical.

In immunology, it is important to describe diverse situations including the course of different types of infectious disease, the development of autoimmune diseases and the destruction of tumour cells by the immune system. It would be unreasonable to expect that a simple mechanism could be the key to describing all these situations. One strategy to try to obtain more understanding is to choose one mechanism and to investigate which types of situations it suffices to describe. This may be done by combining mathematical models with experimental data. What are the restrictions under which the type of model studied in this paper might be appropriate? The first assumption is that in the situation to be explained the distinction between self and non-self takes place within an individual T cell. In other words, it is assumed that it is not necessary to consider the population dynamics of the T cells involved or even the interaction of their population with that of other types of immune cells such as regulatory T cells or dendritic cells. A quite different type of mathematical model, where population effects are considered, can be found in [14]. In that case, in contrast with the lifetime dogma, the response depends on the rate of change of the antigen concentration. The second assumption which is important for the models studied here is that the distinction between self and non-self takes place on a sufficiently short time scale, say three minutes. On longer time scales, there may be essential effects related to the spatial distribution of molecules on the T cell surface (formation of the immunological synapse) so that a description by means of ordinary differential equations may be insufficient. It may also happen that some TCRs become inactive on a longer time scale (limiting signalling model, cf. [6]).

In this paper, we have concentrated on studying the mathematical properties of a particular model for the biological phenomenon of T-cell activation with arbitrary values of the parameters. A complementary question is to what extent known experimental data on the parameters may further constrain the dynamics in this model. In addition, it is important to know whether this model is consistent with all biological data and how it compares to other possible models for the same biological system. For a discussion of this, we refer to [6,13,15]. It was indicated in [6] that the situation where *C*_*N*_ is a decreasing function of *ν*_1_ cannot be reproduced using the model of [4]. Our results indicate that a failure of the model to reproduce this effect must depend not only on the model itself but on the choice of parameters used for simulations. At the same time, it may be that this effect only occurs in experiments where the measurements are done on long time scales (many hours) and not on the time scale of the initial activation (a few minutes) for which the models of [3,4] were primarily intended. We plan to investigate these questions further elsewhere.

## Data accessibility

This article has no additional data.
